# Cardioprotective Effects of PPARβ/δ Activation against Ischemia/Reperfusion Injury in Rat Heart Are Associated with ALDH2 Upregulation, Amelioration of Oxidative Stress and Preservation of Mitochondrial Energy Production

**DOI:** 10.3390/ijms22126399

**Published:** 2021-06-15

**Authors:** Ioanna Papatheodorou, Eleftheria Galatou, Georgios-Dimitrios Panagiotidis, Táňa Ravingerová, Antigone Lazou

**Affiliations:** 1 Laboratory of Animal Physiology, Department of Zoology, School of Biology, Aristotle University of Thessaloniki, 54124 Thessaloniki, Greece; pkioanna@bio.auth.gr (I.P.); eleftheriagala@gmail.com (E.G.); akispanagiwtidis@gmail.com (G.-D.P.); 2 Institute for Heart Research, Centre of Experimental Medicine, Slovak Academy of Sciences, 9 Dúbravská cesta, 84104 Bratislava, Slovakia; tatiana.ravingerova@savba.sk

**Keywords:** ischemia/reperfusion, oxidative stress, ALDH2, PPARβ/δ, mitochondria

## Abstract

Accumulating evidence support the cardioprotective properties of the nuclear receptor peroxisome proliferator activated receptor β/δ (PPARβ/δ); however, the underlying mechanisms are not yet fully elucidated. The aim of the study was to further investigate the mechanisms underlying PPARβ/δ-mediated cardioprotection in the setting of myocardial ischemia/reperfusion (I/R). For this purpose, rats were treated with PPARβ/δ agonist GW0742 and/or antagonist GSK0660 in vivo and hearts were subjected to ex vivo global ischemia followed by reperfusion. PPARβ/δ activation improved left ventricular developed pressure recovery, reduced infarct size (IS) and incidence of reperfusion-induced ventricular arrhythmias while it also up-regulated superoxide dismutase 2, catalase and uncoupling protein 3 resulting in attenuation of oxidative stress as evidenced by the reduction in 4-hydroxy-2-nonenal protein adducts and protein carbonyl formation. PPARβ/δ activation also increased both mRNA expression and enzymatic activity of aldehyde dehydrogenase 2 (ALDH2); inhibition of ALDH2 abrogated the IS limiting effect of PPARβ/δ activation. Furthermore, upregulation of PGC-1α and isocitrate dehydrogenase 2 mRNA expression, increased citrate synthase activity as well as mitochondrial ATP content indicated improvement in mitochondrial content and energy production. These data provide new mechanistic insight into the cardioprotective properties of PPARβ/δ in I/R pointing to ALDH2 as a direct downstream target and suggesting that PPARβ/δ activation alleviates myocardial I/R injury through coordinated stimulation of the antioxidant defense of the heart and preservation of mitochondrial function.

## 1. Introduction

Ischemic heart disease and its most common manifestation acute myocardial infarction (MI) and the development of heart failure, remains a leading cause of death and disability worldwide. The only available treatment of choice up to date is timely and effective myocardial reperfusion. However, instead of providing a salvation, reperfusion can paradoxically intensify injury and cardiomyocyte death, known as ischemia/reperfusion (I/R) injury [1]. Accordingly, novel therapeutic strategies focus on reducing the size of the myocardial infarction while preventing the development of I/R injury and heart failure by enhancing processes aiming at preserving cardiac function. Despite intense efforts to address post-I/R cardiac injury and dysfunction and the identification of important regulators of cardiac homeostasis with potential cardioprotective properties, translation of cardioprotection to clinical practice has proven difficult. Potential reasons may include the complexity of mechanisms underlying I/R or the requirement for synergistic multitarget effects for optimal cardioprotection [2,3].

Myocardial I/R injury is a complex process during which the ability of physiological mechanisms to return the cardiac cells to homeostasis is numbed. A major cause of this is calcium overload which damages cellular components and drains energy (ATP) as ion pumps in the sarcolemma and sarcoplasmic reticulum (SR) are engaged to return cytosolic calcium back to appropriate levels [4]. In addition, rapid restoration of oxygen overwhelms the mitochondria, leading to redox balance deregulation and massive reactive oxygen species (ROS) production, lipid peroxidation and formation of toxic intermediate aldehydes. These toxic products often react with and inactivate subcellular components and further aggravate oxidative damage and overall cardiomyocyte dysfunction [5,6]. Intracellular calcium overload, rapid normalization of pH upon reperfusion and oxidative stress result in the opening of the mitochondrial permeability transition pore (MPTP) triggering cell death [7]. Since it is widely accepted that reperfusion-induced oxidative stress is one of the main players in myocardial I/R injury, pharmacological approaches that seek to ameliorate its effects have shown some promising results and are considered a potentially useful strategy in the management of I/R injury [8,9,10,11].

Over the past years, a wealth of data has shown that peroxisome proliferator activated receptors (PPARs), a family of nuclear receptors (with three members, α, β/δ and γ) that act as ligand-inducible transcription factors, demonstrate pleiotropic cardioprotective properties [12,13,14,15,16]. Besides being the main transcriptional regulators of myocardial lipid metabolism and energy homeostasis, PPARs also exhibit antioxidant and anti-inflammatory properties [17,18]. However, the mechanisms underlying these protective effects are largely unexplored, and it is still a matter of debate whether cardioprotection is attributed to the modulation of cardiac energy metabolism or to antioxidative and anti-inflammatory effects of PPARs. All three PPAR isoforms have been implicated in the modulation of oxidative stress, although different mechanisms may be employed by each of them. Transcriptional regulation of antioxidant genes or other effectors that could modulate oxidative stress and effects on signal transduction pathways have been described [17]. PPARβ/δ activation suppressed ROS generation in adult rat cardiac myocytes subjected to oxidative stress, implicating catalase upregulation [19]. Furthermore, cardiac PPARβ/δ deletion in mice resulted in depressed bioenergetics, cardiac hypertrophy, and congestive heart failure [20], while it has been also shown that PPARβ/δ is essential for the adult heart to maintain mitochondrial capacity and oxidative metabolism [21]. However, the mechanisms underlying the effect of PPARβ/δ activation on antioxidant defense have yet to be fully elucidated.

In the present study we sought to further investigate the mechanism underlying PPARβ/δ-mediated beneficial effects in the setting of myocardial I/R. We used the agonist GW0742 to activate PPARβ/δ and we confirmed transcriptional activation of the receptor by determining the expression of target genes. We hypothesized that mitochondrial ALDH2 and preservation of mitochondrial function can play an important role in the mechanism of cardioprotection. We propose a new mechanism that implicates a coordinated action of PPARβ/δ transcriptional activation on both antioxidant enzymes and mitochondrial function to ameliorate oxidative stress and maintain energy production, resulting in the alleviation of I/R injury.

## 2. Results

### 2.1. Administration of GW0742 Improves Myocardial Recovery and Reduces Infarct Size after I/R

**Figure 1:**
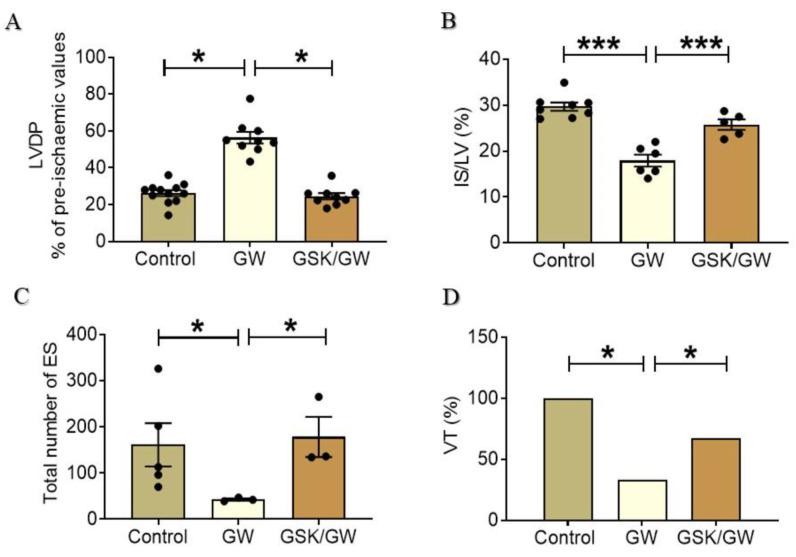
Activation of PPARβ/δ improves cardiac recovery and reduces arrhythmias and infarct size following I/R. (**A**) Left ventricular developed pressure (LVDP, systolic minus diastolic pressure) was measured during equilibration and post-ischemic period; (**B**) Infarct size (IS), expressed in % of risk area (LV). (**C**) Ectopic activity was evaluated as total number of extrasystoles (ES) during the post-ischemic period. (**D**) Incidence of ventricular tachycardia expressed in percentage. Data are presented as mean ± SEM, *n* = 3–12 per group, * *p* < 0.05 and *** *p* < 0.001. GW, pretreated with agonist GW0742; GSK/GW, pretreated with antagonist GSK0660 and agonist GW0742.

Neither GW0742 nor GSK0660 administration affected heart function. No significant differences among the groups were observed in the baseline pre-ischemic values of functional parameters such as heart rate, coronary flow, left ventricular (LV) systolic and end-diastolic pressure (LVSP, LVEDP) and LVDP (LV systolic minus LV diastolic pressure). Pretreatment of animals with the PPARβ/δ ligand GW0742 led to a significant increase in post-ischemic LVDP recovery (Figure 1A) which appeared higher by almost 52% (26 ± 3.5 for the Control group compared with 54.5 ± 10 for the GW group) while administration of the PPARβ/δ-specific antagonist GSK0660 abolished this result and led to a significant decrease of LVDP recovery (40% decrease, 54.5 ± 10 for the GW group compared with the 22.9 ± 2.5 for the GSK/GW group). The above results were also accompanied by a significant reduction in infarct size in the GW0742-treated animal group compared with the Control group (IS/LV: 17.9 ± 1.3% for the GW group compared with the 29.6 ± 0.9% for the Control group—Figure 1B). Again, this effect was attenuated in the presence of PPARβ/δ antagonist GSK0660 (IS/LV: 25.8 ± 1.4% for the GSK/GW group compared with the 17.9 ± 1.3% for the GW group) suggesting that the effect was specific to PPARβ/δ activation. Consistently, administration of GW0742 resulted in a concomitant reduction in overall arrhythmia severity in the hearts of treated animals (Figure 1C) compared with that in the untreated ones (total number of reperfusion-induced ES 161.4 ± 46.7 for the Control group compared with 43 ± 4 for the GW group), which was attenuated in the presence of the PPARβ/δ antagonist (ES 178.3 ± 43.3 for the GSK/GW group). Importantly, more severe tachyarrhythmias (ventricular tachycardia, VT) occurred in each heart of the Control group (100% incidence), whereas in GW-treated group, VT incidence was significantly reduced to 33% (Figure 1D). This antiarrhythmic effect was abrogated by PPARβ/δ antagonist which significantly increased the incidence of VT in the GSK/GW group to 67% as compared with 33% in the GW group.

### 2.2. Administration of GW0742 Results in Activation of PPARβ/δ and Up-Regulation of Its Target Genes Angptl4 and Mcad

**Figure 2:**
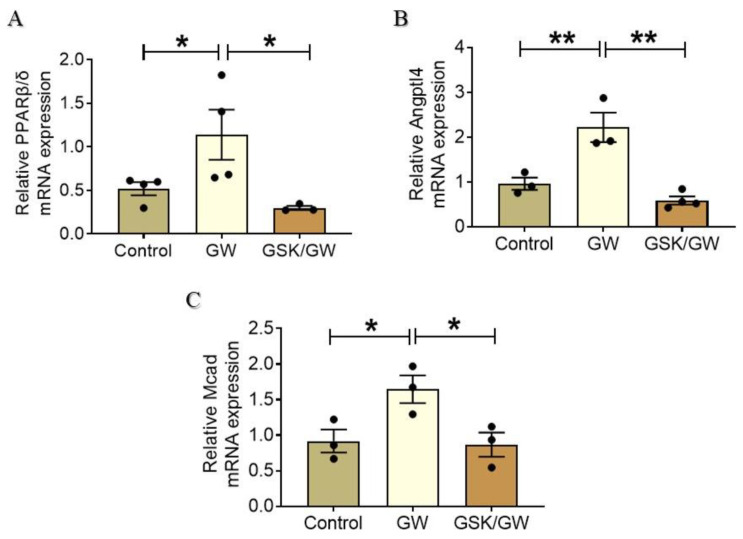
Administration of PPARβ/δ agonist GW0742 results in upregulation of PPARβ/δ and of its target genes Angptl4 and Mcad. Relative mRNA expression of PPARβ/δ (**A**), Angptl4 (**B**) and Mcad (**C**) was normalized to β-actin. Data are presented as mean ± SEM, *n* = 3–4, * *p* < 0.05 and ** *p* < 0.01. GW, pretreated with agonist GW0742; GSK/GW, pretreated with antagonist GSK0660 and agonist GW0742.

To further confirm that PPARβ/δ is activated following administration of its specific ligand GW0742, the expression of PPARβ/δ mRNA as well as that of downstream target genes was determined. Administration of GW0742 resulted in a two-fold increase of PPARβ/δ mRNA expression compared with the Control group while co-administration of the antagonist GSK0660 abolished this effect (Figure 2A). We further examined the expression of PPARβ/δ target genes angiopoietin-like peptide 4 (Angptl4) and medium-chain acyl-CoA dehydrogenase (Mcad). Angptl4 is a multifaceted protein that is involved in maintenance of lipid homeostasis and has been established as PPARβ/δ-specific target in the heart [22,23]. Angptl4 mRNA expression level showed two-fold increase in the GW group returning to baseline at GSK/GW group, thus, further corroborating PPARβ/δ activation in GW group hearts (Figure 2B). Similar expression profile was obtained for Mcad, the enzyme which catalyzes the initial step of the of fatty acids oxidation (FAO) process (Figure 2C). Taken together, these results support the activation of transcriptional activity of PPARβ/δ and its mediated effects after administration of the ligand GW0742.

### 2.3. PPARβ/δ Upregulates Myocardial Antioxidant Enzymes and Ameliorates I/R-Induced Oxidative Stress

**Figure 3:**
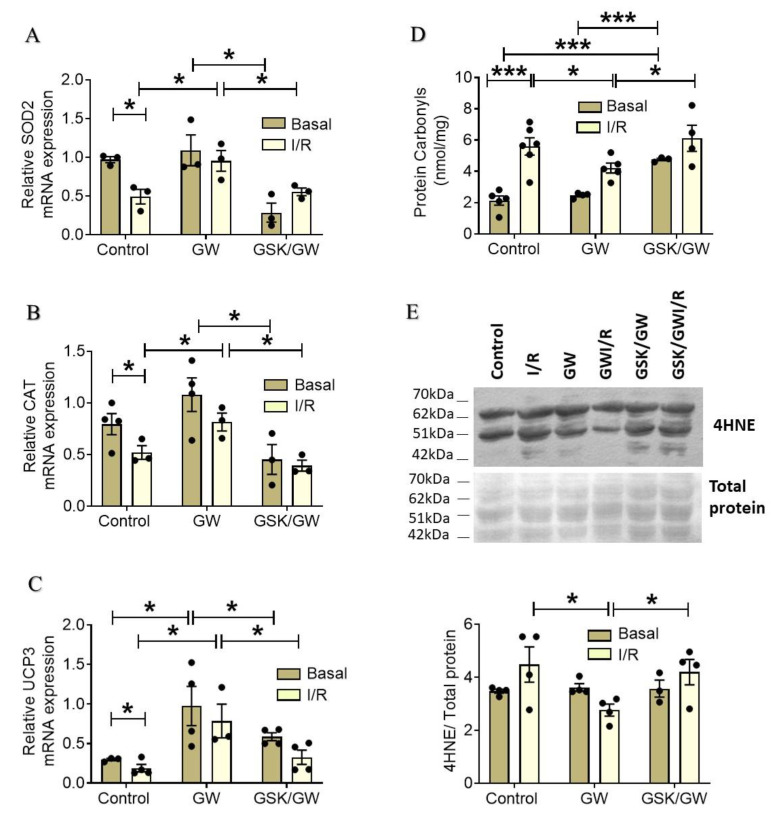
Activation of PPARβ/δ upregulates myocardial antioxidant enzymes SOD2, catalase and UCP3 and ameliorates I/R-induced oxidative stress. Relative mRNA expression of (**A**) mitochondrial SOD2, (**B**) catalase and (**C**) UCP3 was determined by qPCR and normalized to β-actin. (**D**) Quantification of protein carbonyls. (**E**) Representative blot of 4-HNE mediated protein adducts formation (upper panel) and quantification by densitometric analysis (lower panel). Data are shown as mean ± SEM, *n* = 3–5, * *p* < 0.05 and *** *p* < 0.001. GW, pretreated with agonist GW0742; GSK/GW, pretreated with antagonist GSK0660 and agonist GW0742.

Reperfusion of ischemic myocardium is accompanied by massive ROS production causing extensive oxidative stress, which is widely accepted as one of the main mediators behind I/R-injury and cardiac dysfunction. We have previously shown that PPARβ/δ attenuates the generation of ROS in cardiac myocytes subjected to oxidative stress [19]. To examine the effect of PPARβ/δ activation in myocardial I/R, we determined the mRNA expression of the endogenous antioxidant enzymes superoxide dismutase 2 (SOD2) and catalase, as well as uncoupling protein 3 (UCP3), which also functions as a member of the cellular antioxidant defense system that protects against oxidative stress [24]. Administration of GW0742 resulted in increased expression of SOD2 and catalase mRNA in I/R conditions compared with Control I/R while basal expression of the enzymes remained unaffected (Figure 3A,B). On the other hand, a strong up-regulation of UCP3 mRNA expression was observed at basal conditions in GW group in comparison with the respective Control group (Figure 3C). UCP3 mRNA expression was maintained at high level after I/R in GW group, demonstrating almost 4-fold increase compared with the respective Control. Co-administration of the antagonist GSK0660 together with GW0742 led to a significant decrease of mRNA expression of all enzymes under both basal and I/R conditions (Figure 3A–C). Additionally, protein carbonyls and 4-hydroxy-2-nonenal (4-HNE) adducts, as indices of oxidative stress, were decreased (approximately 38% and 31%, respectively) in the GW group and in a PPARβ/δ-dependent manner (Figure 3D,E). Taken together, these results suggest a PPARβ/δ-mediated activation of myocardial defense mechanisms against I/R-induced oxidative stress.

### 2.4. PPARβ/δ Upregulates and Activates ALDH2 in I/R Hearts Resulting in the Reduction of Infarct Size

**Figure 4:**
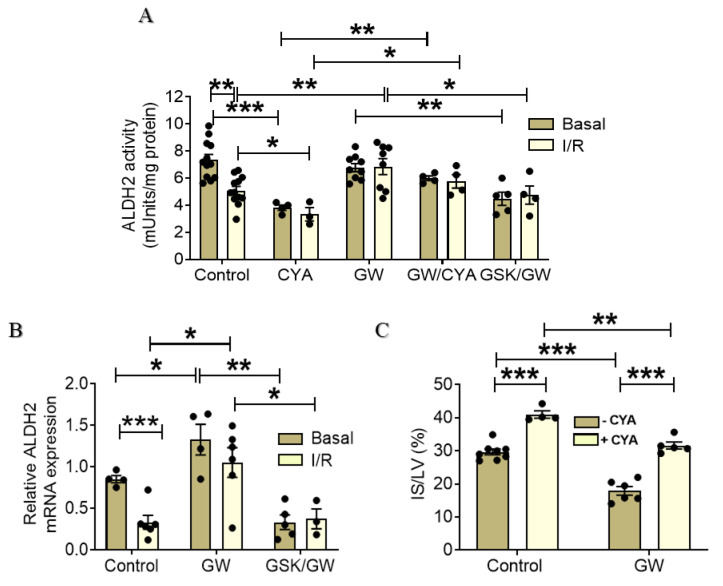
PPARβ/δ activation upregulates mitochondrial ALDH2 activity and expression leading to decreased infarct size after I/R. (**A**) ALDH2 enzymatic activity. Data are shown as mean ± SEM, *n* = 4–12 per group. (**B**) mRNA expression of ALDH2 was determined by qPCR and normalized with β-actin. Data are shown as mean ± SEM, *n* = 4–6 per group. (**C**) Infarct size expressed as % of risk area (LV) was determined in the presence or absence of ALDH2 inhibitor cyanamide (CYA). Data are shown as mean ± SEM, *n* = 5–8 per group. * *p* < 0.05, ** *p* < 0.01 and *** *p* < 0.001. GW, pretreated with agonist GW0742; GSK/GW, pretreated with antagonist GSK0660 and agonist GW0742; CYA, ALDH2 inhibitor cyanamide.

Given the PPARβ/δ-mediated reduction in 4-HNE adducts and the up-regulation of cardiac antioxidant enzymes, we sought to determine the effect of PPARβ/δ activation on the activity of mitochondrial aldehyde dehydrogenase 2 (ALDH2). ALDH2 is best known for its role in the metabolism of endogenous acetaldehyde but also plays a key role in oxidizing aldehydic products such as 4-HNE and malondialdehyde (MDA) that arise from lipid peroxidation under oxidative stress [25,26]. Basal ALDH2 activity did not differ between Control and GW-treated group (Figure 4A). On the other hand, while ALDH2 activity was significantly decreased after I/R in the Control group, it was maintained close to the pre-ischemic value in the GW-treated group (Figure 4A). Once more, this was confirmed to be a PPARβ/δ-mediated effect as increased ALDH2 activity in I/R was abolished in the presence of the antagonist GSK0660. Of note, treatment with GW0742 was able to partially reverse ALDH2 inactivation mediated by cyanamide.

Interestingly, PPARβ/δ activation with the administration of GW0742 resulted in up-regulation of ALDH2 at the level of mRNA under basal conditions implicating ALDH2 as a direct transcriptional target of the receptor (Figure 4B). It is noteworthy that ALDH2 mRNA expression was completely abolished in the presence of PPARβ/δ antagonist to a level below that of control, suggesting a role for the receptor in the basal regulation of ALDH2 expression. Increased ALDH2 mRNA expression level was maintained in the GW group under I/R demonstrating 3-fold increase compared with Control group in I/R (Figure 4B).

To assess the effect of PPARβ/δ-mediated ALDH2 up-regulation on I/R injury, we determined infarct size in the presence of cyanamide, the specific post-translational inhibitor of ALDH2 [27]. Administration of cyanamide before induction of I/R led to a 37% increase in infarct size (IS/LV: 41.0 ± 1.1% for CYA group in comparison with 29.8 ± 1.4% for the Control group) while prior PPARβ/δ activation resulted in a significant attenuation of this effect (IS/LV: 31.9 ± 1.9% for the GW/CYA group compared with 41.0 ± 1.1% for the CYA group). Of note, although the infarct size observed in GW/CYA group was significantly lower than in the CYA group, it was also significantly higher than in the GW group (IS/LV: 17.4 ± 1.3% for the GW group compared with 31.9 ± 1.9% for the GW/CYA group—Figure 4C). Taken together, these results implicate the presence of other, ALDH2-independent but PPARβ/δ-dependent, mechanisms that modulate infarct size under these conditions.

### 2.5. PPARβ/δ-Mediated Improvement of Mitochondrial Energy Production

**Figure 5:**
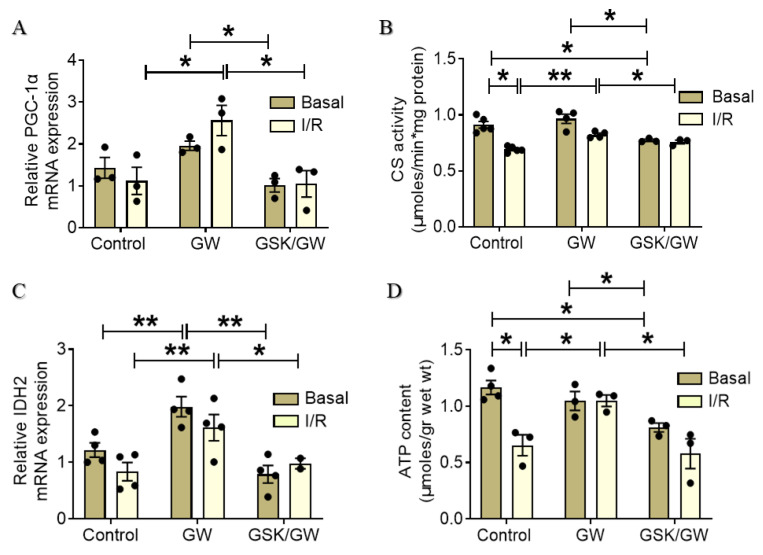
PPARβ/δ activation preserves mitochondrial energy production and function in I/R. (**A**) PGC-1α mRNA expression was normalized to β-actin. (**B**) Citrate synthase activity. (**C**) IDH2 mRNA expression was normalized to β-actin. (**D**) ATP content expressed as μmoles/g of cardiac tissue. Data are shown as mean ± SEM, *n* = 3–5, * *p* < 0.05 and ** *p* < 0.01. GW, pretreated with agonist GW0742; GSK/GW, pretreated with antagonist GSK0660 and agonist GW0742.

I/R-induced oxidative stress and 4-HNE adduct formation are detrimental for mitochondrial function since many components of the mitochondrial energy production machinery, including NADP-dependent isocitrate dehydrogenase (IDH2), are also 4-HNE targets [5]. At the same time, components of the electron transport system are also prone to carbonylation during oxidative stress [28]. These effects along with energy deprivation and mitochondrial homeostasis deregulation, significantly contribute to post-I/R cardiac dysfunction. Consequently, we investigated the effect of PPARβ/δ activation on several components involved in mitochondrial function. Peroxisome proliferator activated receptor gamma co-activator 1 alpha (PGC-1α), a master regulator of mitochondrial biogenesis and of genes involved in mitochondrial energy production [29] showed two-fold increase after I/R in the GW-treated group while this increase was abrogated in the presence of the antagonist GSK0660 (Figure 5A). In parallel, a PPARβ/δ-mediated increase in citrate synthase (CS) activity, an enzyme in the first step of Krebs cycle, which is commonly used as a quantitative marker for the presence of intact mitochondria, was observed in I/R after administration of GW0742 compared with the respective Control group (0.69 ± 0.01 μmoles/min·mg protein for the Control group compared with 0.82 ± 0.01 μmoles/min·mg protein for the GW group) (Figure 5B). Although PPARβ/δ activation did not seem to alter CS activity at basal conditions, inhibition of the receptor led to significantly lower enzyme activity (0.77 ± 0.01 μmoles/min·mg protein for the GSK/GW group compared with 0.967 ± 0.056 μmoles/min·mg protein for the GW group). Furthermore, administration of GW0742 resulted in transcriptional up-regulation of mitochondrial NADP-dependent IDH2, a critical enzyme for the fine regulation of the Krebs cycle activity, both under basal and I/R conditions (Figure 5C). This effect was abrogated in the presence of the inhibitor GSK0660. In line with the above results, ATP content was maintained at high levels in I/R after the administration of GW0742, demonstrating a higher mitochondrial energy production in this group as compared with the respective control. (1.05 ± 0.05 μmoles/g for the GW group and 0.65 ± 0.09 μmoles/g for the Control group) (Figure 5D). Of note, although activation of the receptor did not alter ATP production at basal conditions, co-administration of the antagonist led to decreased ATP content in comparison with both the Control and GW group (1.17 ± 0.06 μmoles/g for the Control group, 1.04 ± 0.08 μmoles/g for the GW group and 0.81 ± 0.04 μmoles/g for the GSK/GW group) implicating a role for PPARβ/δ in the regulation of mitochondrial energy production in the myocardium at basal conditions. Taken together, the above results suggest an overall PPARβ/δ-mediated amelioration of mitochondrial function after I/R leading to preservation of energy production.

## 3. Discussion

MI is still the most frequent cause of heart failure worldwide while no effective treatment has been developed so far. In the present study we demonstrate that nuclear receptor PPARβ/δ activates cardiac antioxidant defense enzymes and improves mitochondrial energy production and function, ultimately leading to reduced infarct size and enhanced cardiac performance in I/R.

Accumulating evidence supports the cardioprotective role of PPARβ/δ under various pathological conditions. Conditional cardiac-specific deletion of PPARβ/δ results in increased cardiac lipid accumulation and cardiomyopathy [20] whereas PPARβ/δ is downregulated in rat heart after I/R [13]. Early studies have shown that PPARβ/δ agonists are cardioprotective in the setting of I/R as manifested by reduced infarct size and improved post-ischemic recovery of contractile function in normal and Zucker fatty rats [30,31]. On the other hand, PPARβ/δ-independent effect of ligands on cardiomyocytes [32] and other cell types [33,34] have been also reported. Here, we further investigate the effect of PPARβ/δ ligand GW0742 in the setting of myocardial I/R and elucidate the underlying mechanisms. In addition to the improvement of post-ischemic contractile function and the reduction of infarct size, activation of PPARβ/δ resulted in a concomitant reduction in overall ectopic activity and incidence of ventricular tachycardia demonstrating beneficial effect also on arrhythmogenesis after I/R (Figure 1). Furthermore, using the specific antagonist GSK0660, we demonstrate that the cardioprotective effects of GW0742 are PPARβ/δ -mediated. Pre-treatment of animals with the specific PPARβ/δ antagonist GSK0660 abolishes the IS-limiting effect of GW0742 as well as its beneficial effect on cardiac recovery and arrhythmogenesis after ex vivo global I/R (Figure 1). The inhibition of PPARβ/δ in the presence of GSK0660 is confirmed by the decreased expression of the receptor itself and downstream target genes (Figure 2).

Oxidative stress is the primary causative factor of myocardial damage and dysfunction in I/R and mitochondria are the main source of ROS [6]. Our own previous data demonstrate the protective effect of PPARβ/δ on cardiomyocytes subjected to oxidative stress that is mediated by suppression of ROS generation and inhibition of apoptosis [19]. In line with these data, administration of GW0742 resulted in amelioration of oxidative stress after myocardial I/R as evidenced by the decreased formation of protein carbonyls and the decreased accumulation of 4-HNE (Figure 3D,E). This effect can be partly attributed to the direct transcriptional upregulation of the antioxidant defense of the heart such as the mitochondrial SOD2, catalase and UCP3 (Figure 3A–C). Transcriptional regulation of antioxidant enzymes by PPARβ/δ in cardiac myocytes has been previously shown [19,21,35,36]. In addition, UCP3, an established target of PPARβ/δ [37] has been shown to play a pivotal role in regulation of mitochondrial function and cell survival in the ischemic heart possibly due to its ability to promote mitochondrial uncoupling and control ROS production [24,38]. Other mechanisms that PPARβ/δ utilizes to regulate the oxidative balance and production of ROS implicate regulation of nitric oxide synthesis and iNOS (inducible NO synthase) expression, reperfusion-induced NFκΒ nuclear translocation and COX-2 activation [31,39].

In animal myocardial I/R models, accumulation of toxic aldehydes, such as 4-HNE, is considered to be an important mechanism for myocardial damage [40]. The 4-HNE act by forming protein adducts on subcellular components among which are various components of the mitochondrial matrix and electron transport system and have been correlated with worsened mitochondrial homeostasis, decreased energy production, and exacerbated cell death [5,40]. ALDH2 is the enzyme responsible for clearance and deactivation of 4-HNE which are rapidly generated and released during reperfusion. ADLH2 is highly expressed in heart and its cardioprotective effects in heart diseases including cardiac I/R injury have been recognized [41,42]. Overexpression of ALDH2 in a mouse model or enhancement of its activity via the small-molecular activator Alda-1 diminished myocardial infarct size, prevented the progression of ventricular remodeling and improved the long-term survival after I/R [25,43,44]. On the other hand, ALDH2 deficiency exacerbated myocardial ischemia/reperfusion injury and hypertension-induced heart failure [43,45]. ALDH2 activity was decreased in rat heart following I/R (Figure 4A), in line with previous reports showing decreased ALDH2 activity following MI [44] and I/R [46]. The decreased ALDH2 enzymatic activity in I/R is attributed to the excessive 4-HNE generation under these conditions, shown in the present study (Figure 3E) and in other reports [43,47], as it is known that 4-HNE also directly inhibits ALDH2, forming a feedback loop [48]. Consistent with the decreased 4-HNE formation, administration of GW0742 alleviated the suppressed ALDH2 activity in I/R (Figure 4A) suggesting that PPARβ/δ activation prevented 4-HNE induced ALDH2 inactivation through inhibition of oxidative stress. However, it appears that this may not the only mechanism underlying the PPARβ/δ-mediated increase in ALDH2 activity. We demonstrate for the first time that ALDH2 can be transcriptionally upregulated in a PPARβ/δ specific manner (Figure 4B). Administration of GW0742 resulted in increased mRNA expression of ALDH2 both under basal conditions and following I/R (Figure 4B) and this effect was abolished in the presence of the specific PPARβ/δ inhibitor GSK0660. The involvement of ALDH2 activation in the protective effect of PPARβ/δ against I/R injury was confirmed using the ALDH2-specific inhibitor cyanamide. Cyanamide partially blunted the IS-limiting effect of PPARβ/δ (Figure 4C), confirming on the one hand the involvement of ALDH2 in the cardioprotective effect of PPARβ/δ following I/R and further suggesting that other mechanisms may also contribute to this effect.

Mitochondria, as the center of energy supply and ROS production, play a crucial role both as targets and drivers of ischemia/reperfusion injury. Mitochondrial function is heavily compromised following I/R mainly because of ATP depletion, metabolic substrate availability/oxidation imbalance and massive ROS production during reperfusion [7,8]. In addition, ROS-induced 4-HNE protein adducts aggravate mitochondrial function, perpetuating ROS production and impairing the function of components of the Krebs cycle such as IDH2, and subunits of respiratory complexes of the electron transport system [49]. Recent evidence implicates ALDH2 in the suppression of mitochondrial ROS production and in normalizing excessive mitophagy during I/R leading to increased cardiomyocyte survival [46,50]. In addition, PPARβ/δ has been implicated in the regulation of mitochondrial function in the heart through upregulation of co-factor PGC-1α. Constitutive activation of PPARβ/δ leads to increased PGC-1α, higher mitochondrial content and, enhanced mitochondrial palmitate and glucose oxidation whereas inducible cardiac-specific knockout of the receptor abolishes these effects [21,36]. Our data demonstrate that PPARβ/δ activation resulted in transcriptional upregulation of PGC-1α and IDH2, the rate limiting step of the Krebs cycle (Figure 5A,C). Although we have not directly measured expression of these proteins, the enhanced citrate synthase activity (Figure 5B) suggests an overall improvement of oxidative metabolism and stimulation of mitochondrial function following I/R. The beneficial effect of PPARβ/δ activation on mitochondrial function and energy production is further supported by the increased myocardial ATP content after I/R observed in the GW-group (Figure 5D).

**Figure 6:**
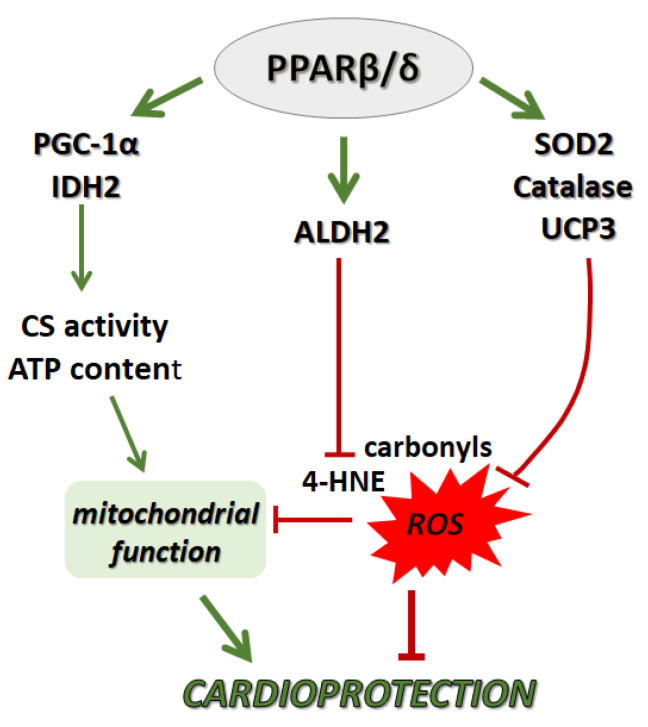
Graphical representation of the proposed effect of PPARβ/δ activation on cardiac antioxidant defense and mitochondrial function during myocardial I/R. PPARβ/δ, peroxisome proliferator activated receptor β/δ; PGC-1α, peroxisome proliferator activated receptor gamma cofactor 1 alpha; IDH2, isocitrate dehydrogenase 2; CS, citrate synthase; ATP, adenosine triphosphate; SOD2, superoxide dismutase 2; CAT, catalase; UCP3, uncoupling protein 3; ALDH2, mitochondrial aldehyde dehydrogenase 2; 4-HNE, 4-hydroxy-2-nonenal.

In conclusion, PPARβ/δ-mediated limitation of infarct size and amelioration of cardiac dysfunction after I/R can be attributed to both activation of multiple antioxidant defense mechanisms and improvement of mitochondrial function. SOD2, CAT and UCP3 may lead to a reduction in ROS production by the mitochondria whereas activation of ALDH2 reduces 4-HNE adduct formation preventing damage of important mitochondrial components. Furthermore, parallel stimulation of PGC-1α, a key regulator of mitochondrial energy metabolism, along with upregulation of enzymes of the Krebs cycle ultimately results in preservation of mitochondrial energy production during I/R (Figure 6). Our findings provide new mechanistic insight into the cardioprotective properties of PPARβ/δ in I/R and underpin the pharmacological modulation of the receptor to effectively manage myocardial I/R injury.

## 4. Materials and Methods

### 4.1. Animals

Animals received proper care in compliance with the ‘‘Guidelines for the Care and Use of Laboratory Animals’’ published by US National Institutes of Health (NIH, Guide, NRC 2011) and the ‘‘Principles of laboratory animal care’’ published by the Greek Government (2013/56) based on EU regulations (2010/63EE). Protocols were approved by the Committee on the Ethics of Animal Experiments of the Directorate of Veterinary Services of Prefecture of Thessaloniki and by the Animal Health and Welfare Division of the State Veterinary and Food Administration of the Slovak Republic. All procedures were performed on male Wistar rats (250–300 g body weight) under general anesthesia, and all efforts were made to minimize suffering. Rats were housed 2 to 4 per cage under standard conditions with a constant 12:12 h light/dark cycle (lights on at 06:0 h) and temperature (22.8 °C ± 2.8 °C), fed a standard diet and had access to tap water ad libitum.

### 4.2. Drug Administration and Experimental Procedures for I/R in Isolated Hearts

Animals were randomly divided in 3 groups according to administration. Control group animals were given intraperitoneal (i.p.) bolus of 1 mL/kg of 10% DMSO and GW group animals were given 1 mg/kg (i.p.) of the specific PPARβ/δ ligand GW0742 (Cayman Chemical, Ann Arbor, MI, USA), 24 h before ex vivo myocardial I/R induction; GSK/GW group animals were given 3 mg/kg i.p. of PPARβ/δ antagonist GSK0660 (Tocris Bioscience, Bristol, UK) and 1 mg/kg of GW0742 30 h and 24 h, respectively, before ex vivo myocardial I/R induction. Dosage for the agonist was based on preliminary experiments and previously published studies [31]; for the antagonist, dosage was based on its characteristics and IC_50_ [51].

**Figure 7:**
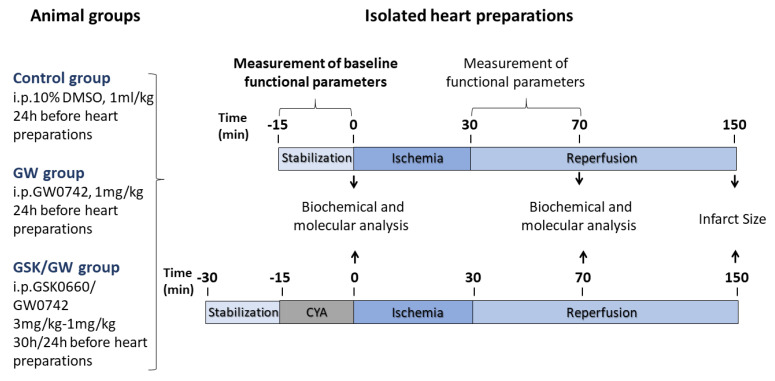
Experimental design for drug administration and induction of ischemia—reperfusion (I/R) in isolated rat hearts. GW, PPARβ/δ-specific ligand GW0742; GSK, PPARβ/δ antagonist GSK0660; CYA, ALDH2 inhibitor cyanamide.

Animals were anesthetized with ketamine/xylazine at 100 mg/kg and 10 mg/kg respectively (Merck, KGaA, Darmstadt, Germany) and given heparin (500 IU, i.p.). Hearts were rapidly excised, placed in ice-cold Krebs-Heisenleit perfusion buffer containing 118.0 mM NaCl, 3.2 mM KCl, 1.2 mM MgSO_4_, 25.0 mM NaHCO_3_, 1.18 mM KH_2_PO_4_, 2.5 mM CaCl_2_ and 10.0 mM glucose. Hearts were cannulated via the aorta and retrogradely perfused in a Langendorff mode at a constant perfusion pressure of 73 mmHg at 37 °C with modified Krebs–Henseleit buffer gassed with 95% O_2_ and 5% CO_2_. Hearts were left to stabilize for 15 min and then were subjected to 30 min of global ischemia by clamping of the aortic inflow. Ischemic period was followed either by a 40 min reperfusion, after which hearts were utilized for molecular analyses, or by a 2 h reperfusion and, measurement of heart function recovery and IS determination. To assess the effect of ALDH2-specific inhibition, hearts from Control and GW groups were perfused with 5 μΜ of cyanamide (CYA, Sigma-Aldrich, St. Louis, MO, USA) for 15 min before ischemia. Cardiac functional parameters were measured during the experimental protocol through continuous registration of an epicardial electrogram (EG) by means of two stainless steel electrodes attached to the apex of the heart and the aortic cannula. Heart rate was calculated from the EG. Left ventricular (LV) pressure was measured by means of a non-elastic water-filled balloon inserted into the LV cavity and connected to a pressure transducer (MLP844, AD Instruments, Spechbach, Germany). Heart function was analyzed using Power Lab/8SP Chart 5 software (AD Instruments, Spechbach, Germany). Post-ischemic recovery of heart function (LVDP, systolic minus diastolic pressure) was expressed as a percentage of the pre-ischemic values. Heart arrhythmias were analyzed using the EG. We focused on the evaluation of reperfusion-induced total ectopic activity (number of extrasystoles) and the incidence of severe ventricular tachycardia (VT). The design of the protocol is summarized in Figure 7.

### 4.3. Determination of IS

The size of the infarcted area was determined with the 2,3,5-triphenyltetrazolium chloride (TTC) method. At the end of reperfusion, hearts were cut into 1–2 mm slices and incubated with 1% TTC in 0.1 Μ phosphate buffer pH = 7.4. Hearts were preserved overnight in PBS buffer (137 mM NaCl, 2.7 mM KCl, 10 mM Na_2_HPO_4_, 1.8 mM KH_2_PO_4_) containing 4% formaldehyde. IS was determined by a computerized planimetric method, described previously [52]. The IS was expressed as a percent of the area at risk, which in the model of global ischemia represents the whole area of the LV.

### 4.4. RNA Preparation and Quantitative Real-Time RT-PCR (qPCR)

Total RNA was extracted with TRIzol reagent (ThermoFisher Scientific, Waltham, MA, USA) according to the manufacturer’s protocol. At the end of the procedure, RNA samples were resuspended in 0.1% (*v*/*v*) diethyl-pyrocarbonate (DEPC)-treated water and were quantified spectrophotometrically at 260 nm at a Quawell Q5000 micro-volume UV-Vis spectrophotometer. Reverse transcription (RT) was performed on 1 μg of total RNA using commercially available cDNA synthesis kit RR037A (Takara BIO, Kusatsu, Japan) in a 20 μL reaction volume as previously described [19]. qPCR analysis was performed using a real-time PCR system (Applied Biosystems, Waltham, MA, USA). Samples were analyzed by the standardized SYBR green method (Kapa Biosystems, Wilmington, MA, USA) according to the manufacturer’s instructions. Each reaction mix contained KAPA SYBR FAST qPCR Master Mix (Kapa Biosystems, Wilmington, MA, USA), 10 pmol of forward or reverse primer and 0.05 μg of cDNA. Gene expression was normalized to β-actin. Relative changes in expression were calculated using the 2 (^−ΔΔCt^) method. Primers for the examined genes are given in Table 1.

### 4.5. Preparation of Whole Cell Protein Extracts

Tissue samples were homogenized in ice-cold lysis buffer containing 20 mM β-glycerophosphate, 50 mM NaF, 2 mM EDTA, 10 mM benzamidine, 20 mM Hepes, 0.2 mM Na_3_VO_4_, 5 mM dithiothreitol (DTT), 0.2 mM leupeptin, 0.01 mM trans-epoxy succinyl-L-leucylamido-(4-guanidino) butane (E64), 0.3 mM phenyl methyl-sulphonyl fluoride (PMSF), 0.12 mM pepstatin and 1% (*w*/*v*) Triton X-100, and extracted on ice for 30 min. Lysates were centrifuged (11,000× *g* for 15 min at 4 °C). Total protein concentration was determined spectrophotometrically using the Biorad assay (BioRad, Hercules, CA, USA).

### 4.6. Western Blot

Protein extracts were boiled in one-third volume of SDS-PAGE sample buffer containing 10% SDS (*w*/*v*), 13% glycerol (*v*/*v*), 300 mM Tris–HCl pH 6.8, 130 mM DTT and 0.2% bromophenol blue (*w*/*v*) and left to migrate on a 10% poly-acrylamide gel containing 375 mM Tris-HCl pH 8.8, 0.275% (*w*/*v*) bis-acrylamide, 0.1% (*v*/*v*) SDS, 0.15% (*w*/*v*) ammonium persulfate and 0.007% (*v*/*v*) TEMED at a constant voltage of 130 V. Proteins were then transferred to a 0.45 mm pore Amersham™ Protran^®^ nitrocellulose membrane (Merck KGaA, Darmstadt, Germany) using a stable voltage of 18 V for 1 h. Non-specific binding sites were blocked with 5% (*w*/*v*) non-fat milk powder in TBST buffer (20 mM Tris–HCl pH 7.5, 137 mM NaCl, and 0.1% (*v*/*v*) Tween 20) for 30 min at room temperature. Membranes were incubated overnight at 4 °C with primary 4-HNE antibody (Abcam, Cambridge, UK) diluted according to the manufacturer’s instructions in TBST buffer containing 1% (*w*/*v*) bovine serum albumin, and then washed in TBST buffer. Membranes were incubated for 60 min at room temperature with horseradish peroxidase-conjugated secondary antibodies (Santa Cruz Biotechnology, Dallas, TX, USA) in TBST buffer containing 1% (*w*/*v*) nonfat milk powder, and they were then washed in TBST buffer. Bands were detected by enhanced chemiluminescence with ECL reagent obtained from Cell Signaling (Beverly, MA, USA) and were quantified by scanning densitometry.

### 4.7. Determination of Protein Carbonyls

Protein carbonylation was determined by 2,4-dinitrophenyl (DNP) hydrazine (2,4-DNPH) derivatization according to Buss et al. [53]. Briefly, frozen tissue samples were lysed with 50 mM sodium phosphate buffer pH 6.7 and centrifuged at 11,000× *g* for 10 min at 4 °C. Part of the supernatant was kept for determination of protein concentration. Samples were reacted with 10 mM of 2,4-DNPH in 2 M HCl for 1 h, deproteinized with 20% TCA and pellets were redissolved in 6 M guanidine hydrochloride. The absorbance was measured at 360 nm and carbonyl content was calculated, using the molar absorption coefficient of 22,000 M^−1^·cm^−1^ relative to protein concentration.

### 4.8. Measurement of ALDH2 and Citrate Synthase Enzymatic Activity

Determination of the enzymatic activity of ALDH2 was based on the spectrophotometric monitoring of the reductive reaction of NAD^+^ to NADH as previously described [25]. Briefly, tissue samples were homogenized in a buffer containing 50 mM Imidazole, 2 mM EDTA and 1 mM MgCl_2_. After centrifugation, the appropriate amount of supernatant was added to 10 mM acetaldehyde in 37.5 mM sodium pyrophosphate buffer, pH = 9.5 and OD was monitored at 340 nm.

For the determination of CS activity, tissue samples were homogenized in imidazole buffer (50 mM imidazole, 2 mM EDTA and 1 mM MgCl_2_) pH = 7.6 and enzymatic activity was determined by spectrophotometric assay in the presence of 25 mM imidazole buffer pH = 7.6, 0.31 mM acetyl-CoA, 0.1 mM DTNB and 0.5 mM oxaloacetate. The reductive reaction of DTNB (Ellman’s reagent) to TNB was measured at 412 nm [54].

### 4.9. Determination of ATP Content

ATP content was determined by spectrophotometric assay as previously described [55]. Briefly, frozen tissue was homogenized with 80 mM perchloric acid (PCA) (1:7 *w*/*v*) and centrifuged at 10,000× *g* for 10 min at 4 °C. The supernatant was neutralized with progressive addition of 3 M KHCO_3_ and re-centrifuged at 15,000× *g* for 10 min at 4 °C. The appropriate volume of supernatant was incubated with 40 mM triethanolamine (TRA) buffer pH 7.6, 3.5 mM MgCl_2_, 0.1% NADP, 5 mM glucose and 50 mU of glucose-6-phosphate dehydrogenase and the reaction was started by the addition of 8 mU hexokinase. The reduction of NADP^+^ to NADPH at 340 nm was monitored over time. ATP concentration was expressed in μmoles/g wet wt.

### 4.10. Statistical Analyses

Statistical significance was evaluated by one-way ANOVA with Student-Newman-Keuls post hoc test using GraphPad Prism (GraphPad Software, San Diego, CA, USA), and was established at *p* < 0.05. Variables with non-parametric distribution (VT incidence) were evaluated using Chi-square test. Data are presented as means ± S.E.M. or percentage of incidence (VT).

## Figures and Tables

**Table 1:** Sequences for the primer pairs used for gene amplification in this study.

Gene Name	Forward Primer	Reverse Primer
*Pparβ/δ*	GAGGGGTGCAAGGGCTTCTT	CACTTGTTGCGGTTCTTCTTCTG
*Angptl4*	GACTTTTCCAGATCCAGCCT	TGATTGAAGTCCACAGAGCC
*Mcad*	CTTCGAGTTGACGGAGCAG	TTGATGAGAGGGAACGGGT
*Sod2*	GGAGTCCAAGGTTCAGGCT	AGGTAGTAAGCGTGCTCCCA
*Catalase*	CCTGTGAACTGTCCCTACCG	ACCCAGTCCCATGCTCTCTC
*Ucp3*	ACAAAGGATTCATGCCCTCC	GATTCCCGCAGTACCTGGAC
*Aldh2*	CAGGTAGCCGAAGGGAACA	GCCAATCGGTACAACAGCC
*Ppargc1a*	AGAGTCACCAAATGACCCCA	GAGTTAGGCCTGCAGTTCCA
*Idh2*	GGACAGTCACCCGCCATTAC	ACATTGCTGAGGCCATGGAT
*β-Actin*	GCCCTGAGGCACTCTTCCA	CGGATGTCCACGTCACACTTC

## Abbreviations

4-HNE	4-hydroxy-2-nonenal
ALDH2	aldehyde dehydrogenase 2
Angptl4	angiopoietin like peptide 4
CS	citrate synthase
CYA	cyanamide
IDH2	isocitrate dehydrogenase 2
I/R	ischemia reperfusion
IS	infarct size
iNOS	inducible NO synthase
IRI	ischemia/reperfusion injury
LVDP	left ventricular developed pressure
Mcad	medium-chain acyl-CoA dehydrogenase
MDA	malondialdehyde
MI	myocardial infarction
MPTP	mitochondrial permeability transition pore
PGC1-α	peroxisome proliferator activated receptor gamma co-activator 1 alpha
PPARβ/δ	Peroxisome Proliferator Activated Receptor β/δ
ROS	reactive oxygen species
SOD2	superoxide dismutase 2
UCP3	uncoupling protein 3
